# Dataset on flammability and functional traits of woody plants in a pine-oak forest of western Mexico

**DOI:** 10.3897/BDJ.13.e173287

**Published:** 2025-12-02

**Authors:** Esthela Rodríguez-García, Emmanuel Sánchez-Gamiño, Moisés Méndez-Toribio

**Affiliations:** 1 Graduate Program, Instituto de Ecología, A. C., Red de Diversidad Biológica del Occidente Mexicano, Centro Regional del Bajío, Pátzcuaro, Michoacán, Mexico Graduate Program, Instituto de Ecología, A. C., Red de Diversidad Biológica del Occidente Mexicano, Centro Regional del Bajío Pátzcuaro, Michoacán Mexico; 2 Secretaría de Ciencia, Humanidades, Tecnología e Innovación (Secihti), Avenida Insurgentes Sur 1582, 03940 Mexico City., Mexico Secretaría de Ciencia, Humanidades, Tecnología e Innovación (Secihti) Avenida Insurgentes Sur 1582, 03940 Mexico City. Mexico; 3 Instituto de Ecología, A. C., Red de Diversidad Biológica del Occidente Mexicano, Centro Regional del Bajío, Pátzcuaro, Michoacán, Mexico Instituto de Ecología, A. C., Red de Diversidad Biológica del Occidente Mexicano, Centro Regional del Bajío Pátzcuaro, Michoacán Mexico

**Keywords:** Darwin Core, fire ecology, fire-prone ecosystems, Trans-Mexican Volcanic Belt, tropical temperate forest.

## Abstract

**Background:**

Plant functional traits provide key information about species’ ecological strategies and their responses to environmental disturbances such as fire. This dataset documents 14 morpho-functional traits of leaves (specific leaf area, leaf water content and leaf dry matter content), stems (maximum height, bark thickness, diameter at 40 cm, wood density, stem water content and stem dry matter content), one regenerative trait (resprouting capacity), as well as fire-related traits (ignition time, flaming time and flammability) and growth form in 50 woody plant species (27 trees, 22 shrubs and one liana) inhabiting a pine-oak forest in the "Barranca del Cupatitzio" National Park (BCNP), located in Uruapan, Michoacán, Mexico. This dataset is formatted according to the Darwin Core Archive standard and is publicly available for use.

**New information:**

This dataset is standardised under the Darwin Core framework. It includes 14 morpho-functional and fire-related traits. The data were obtained from 50 woody species with a diameter at breast height (DBH) > 2.5 cm (27 trees, 22 shrubs and one liana), in a pine-oak forest located in the western Trans-Mexican Volcanic Belt, in the Municipality of Uruapan, Michoacán, Mexico. Here, we report flammability-related traits for these species for the first time. The collection of biological material and the measurement of functional traits followed internationally recognised protocols, ensuring methodological consistency and facilitating integration with other global datasets. The dataset includes values for flammability, ignition time, flaming time, specific leaf area, wood density, stem water and dry matter content, bark thickness, leaf water and dry matter content, maximum height, stem diameter at 40 cm above the ground, plant growth form and resprouting capacity. This information is particularly valuable for studies in functional ecology, ecological restoration, the dynamics of woody plant communities and fire management in temperate, fire-prone ecosystems.

## Introduction

Functional traits are morphological, physiological or phenological characteristics measured at the individual level, referring to inherent attributes of organisms rather than properties of their environment or community context. These traits influence an organism’s performance through their effects on growth, reproduction and survival (Petchey and Gaston 2006, Violle et al. 2007). Functional traits are commonly classified into two categories: “hard traits”, which are directly linked to specific ecological functions (e.g. primary production, nutrient cycling) and typically associated with physiological processes (e.g. photosynthetic rate, water-use efficiency) and “soft traits” which are indirectly related to ecological functions (e.g. specific leaf area, plant height) and are often based on easily measurable morphological attributes (Violle et al. 2007). Traits can also be grouped as response traits, which determine how species respond to environmental drivers, such as climate or disturbance and effect traits, which influence ecosystem processes and functions (Suding et al. 2008). The selection of functional traits depends on the specific goals of the research and should consider the type of organisms, the spatial scales, the environmental gradients involved and the ecological processes under investigation (Petchey and Gaston 2006).

In fire-prone ecosystems, trait-based approaches are valuable for identifying combinations of functional traits that shape species’ responses to fire. The strategies that plants use to cope with fire-related environmental variation determine their capacity to persist under recurrent fire regimes (Pausas 2018). This persistence may occur during, after or between fires and is often mediated by adaptive traits that confer resistance or foster recovery (Vesk 2006, Pausas 2018). Understanding these fire-related traits is particularly relevant in the context of climate change, where increasing fire frequency and intensity may alter ecological assemblages and affect ecosystem vulnerability. Furthermore, datasets like ours are critical for broad-scale analyses, comparative studies and predicting species and community responses to fire across different regions. Functional diversity in fire-affected communities can thus be assessed through traits associated with species’ survival or regeneration following fire disturbance (Pausas 2018). For instance, a thick bark or high wood density provides protection and resistance (Cornelissen et al. 2003) Other traits, such as early leaf senescence or high tissue water content, reduce plant flammability and limit the spread of canopy fires. Additionally, greater maximum height protects reproductive organs by keeping them above the flame zone (Cornelissen et al. 2003). Species with low fire-survival ability may nevertheless persist via post-fire resprouting, a key mechanism for recovery in many fire-adapted systems (Pérez-Harguindeguy et al. 2013, Pausas 2018).

The “Barranca del Cupatitzio” National Park, a Protected Natural Area (PNA) in Uruapan, Michoacán, Mexico, harbours pine and pine-oak forest communities that have been exposed to a range of historical and natural fire regimes over the past 25 years. These ecosystems are representative of temperate tropical forests in the Trans-Mexican Volcanic Belt, which are increasingly vulnerable to shifts in climate and fire regime (Trejo 2008). The Park offers a valuable opportunity to document the functional strategies of woody species under contrasting fire histories within a relatively small, but topographically complex area. Given that similar forest types occur widely across the central and western regions of Mexico (Trejo 2008), the patterns and trait–fire relationships described here can provide predictive insights for other areas facing comparable environmental and management challenges. Moreover, as the Park supports important hydrological and cultural services for the City of Uruapan, improving the ecological understanding of its dominant vegetation contributes directly to regional conservation and restoration planning.

This dataset contributes to a better understanding of the functional diversity of woody species in tropical temperate ecosystems, particularly in relation to their capacity to respond to fire disturbance. By making these data available, we aim to foster the inclusion of fire-related traits in studies of community assembly, species co-existence and ecosystem functioning. Furthermore, published data can provide information for conservation planning and support the design of ecological restoration strategies that are grounded in plant functional traits and adaptive response to disturbance. Functional trait datasets, supported by global databases and theoretical frameworks, are key tools for restoration, conservation and ecological prediction. Their rigorous selection and application enhance function-based management and ecosystem resilience (Petchey and Gaston 2006, Trejo 2008, Pausas 2018).

## General description

### Purpose

Data were obtained through field sampling and laboratory measurements following standardised protocols for functional traits (Cornelissen et al. 2003, Jaureguiberry et al. 2011, Pérez-Harguindeguy et al. 2013). Fourteen traits were measured in 182 individuals belonging to 50 woody plant species (5 gymnosperms and 45 magnoliophytes) with a DBH ≥ 2.5 cm, representing over 75% of the woody community at the study site (Méndez-Toribio et al. 2024). These were primarily soft response traits related to key physiological functions, such as water balance and leaf and wood economic spectrum, as well as hard traits such as flammability, ignition time and sustainability time (Díaz et al. 2015). Live plants were sampled along most of the elevational gradient of the study site from 1724 to 2093 m above sea level. The dataset was formatted as a Darwin Core Archive (DwC-A; Darwin Core Maintenance Group (2021) and includes a core file (occurrence.txt) and an extension file (measurementOrFact.txt) containing individual-level trait data.

## Sampling methods

### Sampling description

Sun-exposed samples of branches, stems and leaves were collected from at least five individuals per species during four sampling dates: in November 2023 and in July, August and October 2024. Sampling was conducted at a consistent height across all individuals, avoiding tissues with visible signs of herbivory. For rare species (e.g. *Alnus acuminata* Kunth, *Cestrum tomentosum* L. f., *Desmodium sumichrastii* (Schindl.) Standl., *Lippia umbellata* Cav., *Ilex brandegeeana* Loes., *Ficus pringlei* S. Watson) or tall species exceeding 10 m in height (e.g. *Pinus devoniana* Lindl., and *Pinus pseudostrobus* Lindl.), samples were obtained from one or two individuals. The biological samples were labelled, wrapped in moistened paper, sealed in plastic bags to prevent dehydration and transported in coolers (Pérez-Harguindeguy et al. 2013) to the Soil laboratory and the Wood and Forest laboratory at the Facultad de Agrobiología, Universidad Michoacana de San Nicolás de Hidalgo. Trait measurements were performed on the same day of collection to minimiseise water loss. Whenever possible, all traits were measured from the same individuals to reduce sampling impact and limit intraspecific variation.

### Quality control

Taxonomic validation was performed through comparison with reference specimens from the IEB Herbarium (Instituto de Ecología, A.C., Centro Regional del Bajío, Pátzcuaro, Michoacán), previously collected in the study area and by cross-referencing with specialised literature. Trait collection, processing and measurement followed standardised protocols developed by Cornelissen et al. (2003), Jaureguiberry et al. (2011) and Pérez-Harguindeguy et al. (2013).

### Step description

The growth form was determined by direct observation. Specific leaf area (SLA) was estimated from five fully sun-exposed, undamaged leaves per individual, collected from equivalent branch heights. Fresh leaves without petioles were weighed and scanned using an Epson^®^ V39 II flatbed scanner at a minimum resolution of 300 dpi. Leaf area (cm^2^) was calculated using ImageJ software (Abràmoff et al. 2008). SLA was then computed as the ratio of leaf area to dry mass (Cornelissen et al. 2003, Pérez-Harguindeguy et al. 2013). For pine needles, length and width were measured using a Mitutoyo digital caliper (model 530-101) and the area was estimated as 2× length×width (Pérez-Harguindeguy et al. 2013). In the case of compound leaves, SLA was calculated using the smallest photosynthetic unit (i.e. the leaflet). Maximum plant height (H_max_) was recorded with a Nikon Forestry Pro^®^ laser hypsometer. Stem diameter at 40 cm above ground level was measured using a Forestry Suppliers 283D diameter tape.

Leaf and branch dry matter content were calculated as the ratio of dry mass (mg) to fresh mass (g; Pérez-Harguindeguy et al. (2013)). Leaf samples were oven-dried at 70°C and wood samples at 90°C for 72 hours, using a Quincy Lab GC natural convection oven. Mass was measured with an Ohaus analytical balance (model PR224/E). Water content in leaves and branches was estimated as 1000 minus the corresponding dry matter content and expressed in mg/g (Pérez-Harguindeguy et al. 2013).

Bark thickness was measured at five equidistant points around the stem at 40 cm above the ground (D40) using a HAGLOF bark gauge (50 mm). Wood density (g/cm^3^) was estimated from debarked branch segments measuring 2.5 cm in length and 1.5 to 3 cm in diameter, following the water displacement method (Cornelissen et al. 2003, Pérez-Harguindeguy et al. 2013).

Resprouting capacity was evaluated for each species within a network of permanent plots established in 2018 (Méndez-Toribio et al. 2024). For this, 5 - 10 adult individuals per species (DBH ≥ 2.5 cm) that experienced complete aboveground biomass loss due to wildfire were monitored. The proportion of individuals that resprouted was recorded one year after the fire.

Flammability was assessed on fresh terminal portions of secondary branches, 50 cm in length, maintaining their natural architecture, following the protocol described by Jaureguiberry et al. (2011). This method involved a low-tech device in which branches samples were preheated and ignited under standardised conditions. The following components were recorded:

a) **Maximum temperature (MT)** reached during combustion (°C), measured with an Eventek^®^ infrared thermometer (-50°C ~ 550°C) positioned 50 cm from the ignition point. Five readings were taken immediately after the ignition point and the highest value was recorded.

b) **Burning rate (BR)**, calculated as the ratio between the length of burned portion of the sample (cm) and the time required for combustion (s). This metric indicates the rate at which flames spread through the plant material and reflects the species´potential to propagate fire.

c) **Biomass consumed (BC)**, was visually estimated using a six-point ordinal scale: 1 = < 1%, 2 = 1-10%, 3 = 11-25%, 4 = 26-50%, 5 = 51-75% and 6 = 76-100%. This variable estimates the proportion of plant material consumed during combustion.

All component values were standardised on a proportional scale, with 1 representing the highest observed value across all species. The reference values used for standardisation were: MT = 512°C, BR = 0.78 cm/s and BC= 5. The standardised scores of each component were then summed to compute a composite flammability index ranging from 0 (no flammability) to approximately 3 (maximum flammability).

Ignition time (IT) and flame sustainability (FS) were measured on the same samples. It was defined as the time elapsed from placement of the sample in the flammability device until the appearance of a sustained flame (Valette 1990) and was recorded with a digital stopwatch (REESE^®^ RS1060). FS was defined as the duration from ignition to flame extinction (Valette 1990). Mean values of IT and FS were calculated across replicates for each species.

## Geographic coverage

### Description

**Figure 1.**
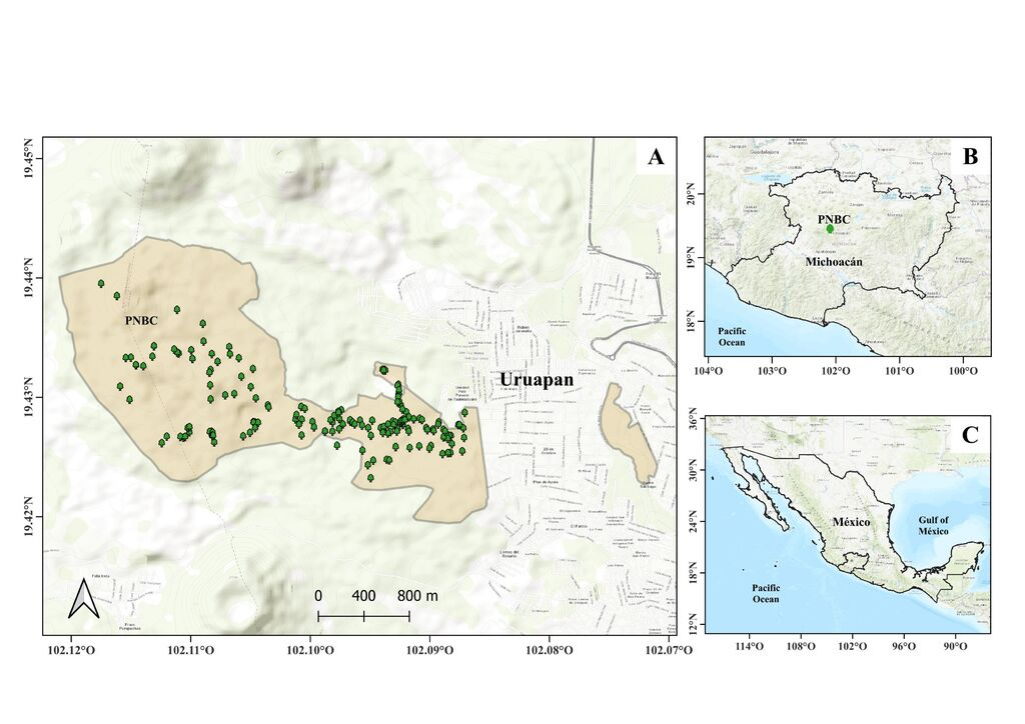
**A** Location of the “Barranca del Cupatitzio” National Park (PNBC) in Uruapan and occurrence points of the sampled species; **B** Mesoscale location of the collection site within the Michoacán State (green point); **C** Macroscale location of the collection site within Mexico.

Biological material was collected in the montane area of the “Barranca del Cupatitzio” National Park, a federally protected area managed by the Comisión Nacional de Áreas Naturales Protegidas (CONANP). The Park spans 439 hectares (Méndez-Toribio et al. 2024) and is located in the west-central region of Michoacán, approximately 130 km from Morelia, the state capital. It lies adjacent to the urban zone of the City of Uruapan (Fig. 1).

It is important to highlight that the selected species are key components of temperate ecosystems (pine forest, oak forest and mixed pine–oak forests) of the Trans-Mexican Volcanic Belt. Additionally, they are widely distributed across Mexico and the American continent (Table 1).

### Coordinates

19.4198° and 19.4401° Latitude; -102.1278° and -102.0722° Longitude.

## Taxonomic coverage

### Description

The taxonomic coverage includes 50 woody species (5 gymnosperms and 45 magnoliophytes), representing 29 botanical families (Anacardiaceae, Apiaceae, Aquifoliaceae, Araliaceae, Asteraceae, Betulaceae, Cannabaceae, Clethraceae, Clusiaceae, Ericaceae, Fabaceae, Fagaceae, Garryaceae, Lauraceae, Moraceae, Namaceae, Oleaceae, Onagraceae, Papaveraceae, Pinaceae, Polygalaceae, Primulaceae, Proteaceae, Rhamnaceae, Rosaceae, Scrophulariaceae, Solanaceae, Verbenaceae and Vitaceae) and 41 genera (*Acaciella*, *Ageratina*, *Alnus*, *Arbutus*, *Baccharis*, *Bocconia*, *Buddleja*, *Calliandra*, *Ceanothus*, *Cestrum*, *Chromolaena*, *Clethra*, *Clusia*, *Dalea*, *Desmodium*, *Enantiophylla*, *Ficus*, *Fraxinus*, *Fuchsia*, *Garrya*, *Ilex*, *Indigofera*, *Inga*, *Oreopanax*, *Lippia*, *Macadamia*, *Monnina*, *Myrsine*, *Persea*, *Pinus*, *Prunus*, *Quercus*, *Rhus*, *Roldana*, *Rumfordia*, *Solanum*, *Trema*, *Vachellia*, *Verbesina*, *Vitis* and *Wigandia*). Geographic coordinates are provided for all recorded species occurrences.

### Taxa included

**Table:** 

Rank	Scientific Name	
species	*Acaciella angustissima* (Mill.) Britton & Rose	
species	*Ageratina areolaris* (DC.) D.Gage ex B.L.Turner	
species	*Ageratina mairetiana* (DC.) R.M.King & H.Rob	
species	*Alnus acuminata* Kunth	
species	*Arbutus xalapensis* Kunth	
species	*Baccharis heterophylla* Kunth	
species	*Bocconia arborea* S. Watson	
species	*Buddleja parviflora* Kunth	
species	*Calliandra grandiflora* (L'Hér.) Benth.	
species	*Ceanothus caeruleus* Lag.	
species	*Cestrum tomentosum* L. f.	
species	*Chromolaena collina* (DC.) R.M.King & H.Rob.	
species	*Clethra hartwegii* Britton	
species	*Clusia salvinii* Donn.Sm.	
species	*Dalea leucostachya* A.Gray	
species	*Desmodium sumichrastii* (Schindl.) Standl.	
species	*Enantiophylla heydeana* J.M. Coult. & Rose	
species	*Ficus pringlei* S. Watson	
species	*Fraxinus uhdei* (Wenz.) Lingelsh	
species	*Fuchsia cylindracea* Lindl.	
species	*Garrya longifolia* Rose	
species	*Ilex brandegeeana* Loes.	
species	*Indigofera thibaudiana* D. C.	
subspecies	*Inga vera eriocarpa* (Benth.) J.León	
species	*Oreopanax peltatus* Linden ex Regel	
species	*Lippia umbellata* Cav.	
species	*Macadamia integrifolia* (Sw.) R.Br. ex Roem. & Schult.	
species	*Monnina ciliolata* Moc. & Sessé ex DC.	
species	*Myrsine coriacea* (Sw.) R.Br. ex Roem. & Schult.	
species	*Persea americana* Mill.	
species	*Pinus devoniana* Lindl.	
species	*Pinus douglasiana* Martínez	
species	*Pinus lawsonii* Roezl ex Gordon	
species	*Pinus leiophylla* Schiede ex Schltdl. & Cham.	
species	*Pinus pseudostrobus* Lindl.	
species	*Prunus serotina* Ehrh.	
species	*Quercus castanea* Née	
species	*Quercus obtusata* Bonpl.	
species	*Rhus aromatica* Aiton	
species	*Roldana chapalensis* (S.Watson) H.Rob. & Brettell	
species	*Roldana michoacana* (B.L.Rob.) H.Rob. & Brettell	
species	*Rumfordia floribunda* DC.	
species	*Solanum chrysotrichum* Schltdl.	
species	*Solanum ferrugineum* Jacq.	
species	*Solanum nigricans* M. Martens & Galeotti	
species	*Trema micranthum* (L.) Blume	
species	*Vachellia pennatula* (Schltdl. & Cham.) Seigler & Ebinger	
species	*Verbesina fastigiata* B.L.Rob. & Greenm.	
species	*Vitis tiliifolia* Humb. & Bonpl. ex Schult.	
species	*Wigandia urens* (Ruiz & Pav.) Kunth	

## Temporal coverage

**Data range:** 2023-11-05 – 2023-11-30; 2024-8-05 – 2024-10-21.

### Notes

Functional trait measurements were conducted in two periods: from 5 to 30 November 2023 and from 5 August to 21 October 2024. Each individual was sampled only once and the exact measurement date is recorded in the "eventDate" field.

## Usage licence

### Usage licence

Open Data Commons Attribution License

### IP rights notes

This dataset is published under the Creative Commons Attribution 4.0 International License (CC-BY 4.0), which permits use, distribution and reproduction in any medium, provided the original authors are properly credited.

## Data resources

### Data package title

Functional traits related to fire in woody species from Barranca del Cupatitzio National Park

### Resource link

https://doi.org/10.15468/46f8xe

### Number of data sets

2

### Data set 1.

#### Data set name

occurrence.txt

#### Data format

Darwin Core

**Data set 1.** 

Column label	Column description
id	Unique identifier for each occurrence.
institutionID	The identifier for the institution having custody of the specimens.
institutionCode	Full name of the institution having custody of the specimens.
basisOrRecord	"HumanObservation" for all records.
occurrenceID	Unique identifier of the biological record.
eventDate	The date during which the record occurred.
language	Spanish (es).
country	The name of the country in which the location occurs.
countryCode	The standard code for the country in which the location occurs
stateProvince	Location refers to the administrative division of Mexico.
municipality	Location refers to the administrative division of Mexico.
verbatimElevation	The elevation (sea level).
decimalLatitude	The geographic latitude in decimal degrees.
decimalLongitude	The geographic longitude in decimal degrees.
scientificName	The name of species or taxon of the occurrence record.
acceptedNameUsage	The currently accepted scientific name for each taxon.
kingdom	The scientific name of the kingdom in which the taxon is classified.
phyllum	The scientific name of the phyllum in which the taxon is classified.
class	The scientific name of the class in which the taxon is classified.
order	The scientific name of the order in which the taxon is classified.
family	The scientific name of the family in which the taxon is classified.
genus	The scientific name of the genus in which the taxon is classified.
specificEpithet	The name of the first or species epithet of the “scientificName".
infraspecificEpithet	Epithet used to identify a taxon below the species level, such as subspecies.
taxonRank	The taxonomic rank of the most specific name in the “scientificName".
scientificNameAuthorship	The authorship information for the “scientificName” formatted according to the conventions of the applicable nomenclatural Code.
taxonomicStatus	Indicates the taxonomic status of the name provided: "accepted", "synonym".

### Data set 2.

#### Data set name

measurementorfacts.txt

#### Data format

Darwin Core

**Data set 2.** 

Column label	Column description
id	Unique identifier matching the id field from the core dataset ocurrence.txt.
measurementID	Unique identifier for the measurement.
measurementType	Name of the measured trait.
measurementValue	Value of the measured trait.
measurementUnit	Units of the measured trait.
measurementDeterminedBy	Name of the person who determined the measurementValue.
measurementDeterminedDate	Date on which the measurement was taken.
measurementMethod	Reference (URL) of the method used to obtain the measurement.
measurementRemarks	Indicates whether the petiole was included in leaf measurements and whether wood trait measurements were performed on branches.

## Additional information

**Conclusion**: This dataset provides standardised trait and flammability measurements of woody species from a tropical-temperate pine-oak forest in western Mexico. The data contribute to the characterisation of functional strategies related to fire response and can support comparative analyses across regions and vegetation types. These data are intended to facilitate integration into broader trait-based and fire ecology research frameworks.

**Conflict of interest**: The authors declare no conflicts of interest.

## Figures and Tables

**Table 1.** Native distribution range of the selected species, based on data from POWO (2025) and Bánki et al. (2025).

**Species**	**Distribution in Mexico**	**Native range of distribution**
*Acaciella angustissima* (Mill.) Britton & Rose	Throughout Mexico	Central and South U.S.A. to Northwest Argentina.
*Ageratina areolaris* (DC.) D.Gage ex B.L.Turner	Throughout Mexico	Mexico to Guatemala.
*Ageratina mairetiana* (DC.) R.M.King & H.Rob	Throughout Mexico	Mexico to El Salvador.
*Alnus acuminata* Kunth	Throughout Mexico	Mexico to North Argentina.
*Arbutus xalapensis* Kunth	Throughout Mexico	South New Mexico to West Texas and Nicaragua
*Baccharis heterophylla* Kunth	Throughout Mexico	Mexico to Guatemala
*Bocconia arborea* S. Watson	Throughout Mexico	Mexico to Central America
*Buddleja parviflora* Kunth	Throughout Mexico	Mexico to Guatemala
*Calliandra grandiflora* (L'Hér.) Benth.	Throughout Mexico	Mexico to Honduras
*Ceanothus caeruleus* Lag.	Throughout Mexico	Mexico to Central America
*Cestrum tomentosum* L. f.	Throughout Mexico	Mexico to Venezuela and Peru
*Chromolaena collina* (DC.) R.M.King & H.Rob.	Throughout Mexico	Mexico to Central America
*Clethra hartwegii* Britton	Mexico Central, Mexico Northeast, Mexico Northwest, Mexico Southwest	Mexico
*Clusia salvinii* Donn.Sm.	Throughout Mexico	Mexico to Central America
*Dalea leucostachya* A.Gray	Throughout Mexico, except Veracruz	Mexico
*Desmodium sumichrastii* (Schindl.) Standl.	Mexico Central, Mexico Southeast, Mexico Southwest	Central and South Mexico
*Enantiophylla heydeana* J.M.Coult. & Rose	Mexico Central, Mexico Northeast, Mexico Northwest, Mexico Southwest	Mexico to Honduras
*Ficus pringlei* S. Watson	Mexico Northeast, Mexico Southwest	Southwest Mexico (to Zacatecas)
*Fraxinus uhdei* (Wenz.) Lingelsh	Throughout Mexico	Mexico to Central America
*Fuchsia cylindracea* Lindl.	Mexico Central, Mexico Southwest	Mexico
*Garrya longifolia* Rose	Mexico Central, Mexico Northeast, Mexico Southwest	Mexico
*Ilex brandegeeana* Loes.	Throughout Mexico, except Veracruz	Mexico to El Salvador
*Indigofera thibaudiana* D. C.	Throughout Mexico	Mexico to Honduras
*Inga vera subsp. eriocarpa* (Benth.) J.León	Throughout Mexico	Mexico
*Oreopanax peltatus* Linden ex Regel	Throughout Mexico, except Veracruz	Mexico to Honduras
*Lippia umbellata* Cav.	Throughout Mexico	Mexico to Central America
*Macadamia integrifolia* Maiden & Betche	Introduced	Southeast Queensland to Northeast New South Wales
*Monnina ciliolata* Moc. & Sessé ex DC.	Mexico Central, Mexico Gulf, Mexico Northeast, Mexico Northwest, Mexico Southwest	Mexico
*Myrsine coriacea* (Sw.) R.Br. ex Roem. & Schult.	Throughout Mexico	Mexico to Tropical America
*Persea americana* Mill.	Mexico Central, Mexico Gulf, Mexico Southeast, Mexico Southwest	Central Mexico to Costa Rica
*Pinus devoniana* Lindl.	Throughout Mexico	Mexico to Guatemala
*Pinus douglasiana* Martínez	Mexico Central, Mexico Gulf, Mexico Northeast, Mexico Southwest	Mexico
*Pinus lawsonii* Roezl ex Gordon	Mexico Central, Mexico Gulf, Mexico Southwest	Central and Southwest Mexico (to Veracruz)
*Pinus leiophylla* Schiede ex Schltdl. & Cham.	Mexico Central, Mexico Gulf, Mexico Northeast, Mexico Northwest, Mexico Southwest	Southeast Arizona to Southwest. New Mexico and Mexico
*Pinus pseudostrobus* Lindl.	Throughout Mexico	Mexico to Honduras
*Prunus serotina* Ehrh.	Throughout Mexico	West Canada to Northwest. U.S.A., East Canada to Guatemala
*Quercus castanea* Née	Throughout Mexico	Mexico to Honduras
*Quercus obtusata* Bonpl.	Mexico Central, Mexico Gulf, Mexico Northeast, Mexico Southwest	Mexico
*Rhus aromatica* Aiton	Mexico Central, Mexico Gulf, Mexico Northeast, Mexico Northwest, Mexico Southwest	North America
*Roldana chapalensis* (S.Watson) H.Rob. & Brettell	Mexico Central, Mexico Northeast, Mexico Northwest, Mexico Southwest	Mexico
*Roldana michoacana* (B.L.Rob.) H.Rob. & Brettell	Mexico Central, Mexico Northeast, Mexico Southwest	Mexico
*Rumfordia floribunda* DC.	Mexico Central, Mexico Gulf, Mexico Northeast, Mexico Northwest, Mexico Southwest	Mexico
*Solanum chrysotrichum* Schltdl.	Mexico Central, Mexico Gulf, Mexico Northeast, Mexico Southeast, Mexico Southwest	Mexico to Central America
*Solanum ferrugineum* Jacq.	Mexican Pacific Is., Mexico Gulf, Mexico Northeast, Mexico Northwest, Mexico Southwest	Mexico and Costa Rica
*Solanum nigricans* M.Martens & Galeotti	Throughout Mexico	Mexico to Central America
*Trema micranthum* (L.) Blume	Throughout Mexico	Tropical and Subtropical America
*Vachellia pennatula* (Schltdl. & Cham.) Seigler & Ebinger	Throughout Mexico	Mexico to Venezuela and Peru
*Verbesina fastigiata* B.L.Rob. & Greenm.	Throughout Mexico	Mexico
*Vitis tiliifolia* Humb. & Bonpl. ex Schult.	Throughout Mexico	Mexico to Venezuela and Ecuador, Caribbean
*Wigandia urens* (Ruiz & Pav.) Kunth	Throughout Mexico	Mexico to Trinidad and Peru
